# A novel region of the insulin B9–23 sequence is targeted by a HIP-reactive diabetogenic T cell clone

**DOI:** 10.3389/fimmu.2026.1837267

**Published:** 2026-07-29

**Authors:** Janet M. Wenzlau, Kaitlin Mangold, Kelli Nicholson, Mylinh Dang, Anita C. Hohenstein, Kelly S. Douglas, James E. DiLisio, Roger Powell, Thomas Delong, Kathryn Haskins, Rocky L. Baker

**Affiliations:** 1 Department of Immunology and Microbiology, University of Colorado School of Medicine, Aurora, CO, United States; 2 Department of Pharmaceutical Sciences, University of Colorado Skaggs School of Pharmacy and Pharmaceutical Sciences, Aurora, CO, United States

**Keywords:** autoimmunity, CD4 T cells, hybrid insulin peptides (HIPs), insulin B-chain, NOD mouse model, type 1 diabetes

## Abstract

Type 1 Diabetes (T1D) is an organ-specific autoimmune disease in which T cells are major drivers, leading to the destruction of beta-cells and insulin deficiency. Although the B-chain of insulin is a critical target for islet-reactive CD4 T cells, the native sequence is poorly antigenic and *in vivo*, insulin elicits only a weak Th1 response. We previously reported that post-translational modification of proinsulin through hybrid insulin peptide (HIP) formation leads to the synthesis of potent epitopes for islet-reactive CD4 T cells, and that cathepsin D (catD), a protease present in beta-cells, can mediate the formation of HIPs through a transpeptidation process. In the present study, we defined the specificity of catD for the insulin B-chain and show a high specificity for proteolytic cleavage following the disease-critical tyrosine 16 residue. We therefore hypothesized that HIP formation occurs at this amino acid. We designed a panel of HIPs containing insB_9–16_ fragments linked to different secretory granule proteins and challenged splenocytes isolated from diabetic NOD mice with insB_9–16_ HIPs; ELISPOT analysis showed elevated inflammatory responses to several peptides. Among this panel of HIPs, we identified the 2.40HIP, a fusion peptide between insB_9–16_ and the C-peptide, as a potent epitope recognized by PD12-2.40, a diabetogenic CD4 T cell clone isolated from the islets of a 12-week-old NOD mouse. These results define a new antigenic region of the insulin B:9–23 peptide and demonstrate that this sequence of the B-chain becomes dramatically more antigenic when post-translationally modified through HIP formation.

## Introduction

Type 1 diabetes (T1D) is an autoimmune disease in which autoreactive T cells selectively destroy insulin-producing pancreatic β-cells in the islets of Langerhans where proinsulin is an important antigen. In the non-obese diabetic (NOD) mouse, a spontaneous model for T1D, the B-chain of insulin is one of the primary targets of both CD4 and CD8 T cells. Whereas both CD4 and CD8 T cells can react to the insB_15–23_ sequence (1, 2), it has been reported that CD4 T cells recognize three additional registers within insB_9–23_ in the context of I-A^g7^: register 1 (insB_12–20_ recognized by “type B” insulin-reactive T cells such as 9B9 T cells); register 2 (insB_13–21_ recognized by “type A” insulin-reactive T cells such as IIT-3 T cells) (3); and register 3 (insB_14-22_), the binding of which is unfavorable due to the presence of an arginine at position 22 (R22). Yet all three registers of native insulin B_9–23_ are poorly antigenic and high concentrations (generally in the micromolar range) of peptide are required to elicit a response by insulin-reactive CD4 T cells. We have hypothesized that the antigenicity of the insulin B_9–23_ sequence *in vivo* could be due to post-translational modification arising from β-cell inflammatory stress. Given the high societal burden and the increasing incidence of T1D worldwide, it is critically important to better understand the contribution to disease of pathogenic insulin-reactive T cells, including the extent and nature of antigenic epitopes recognized by islet-infiltrating CD4 T cells reacting to the insulin B-chain.

We have previously described a novel post-translational modification (PTM) whereby fragments of proinsulin are covalently linked to natural cleavage products of secretogranule proteins through a transpeptidation process leading to the formation of hybrid insulin peptides (HIPs) (4). For example, the ligand of the well-characterized diabetogenic T cell clone BDC-2.5 is formed when the proinsulin C-peptide fragment insC_20–26_ is covalently linked to the peptide WE14, a natural cleavage product of Chromogranin A (ChgA). The resulting 2.5HIP is a robust ligand for the BDC-2.5 T cell clone, requiring only low nanomolar concentrations of peptide to elicit strong T cell responses. Importantly, the 2.5HIP peptide has been identified in the islets of NOD mice (4). Also of note, NOD mice deficient for ChgA, and thus the 2.5HIP, are protected from autoimmune diabetes (5). Other HIPs such as the 6.9HIP and HIP11, which are ligands for the diabetogenic T cell clones BDC-6.9 and NY4.1 respectively, not only have been found in the islets of NOD mice (4, 6, 7) but also in the immunopeptidome eluted from I-A^g7^ (the NOD MHC class II) indicating that these HIPs are presented by islet-infiltrating antigen-presenting cells (APCs) (2). Formation of HIPs containing C-peptide fragments can involve cleavage of proinsulin by cathepsin D (CatD), an aspartic protease present in the secretory granules of β-cells (8), and HIP11, one of the antigenic ligands for the diabetogenic NY4.1 TCR (9), can be formed by this protease *in vitro (*8). These HIPs, all derived from C-peptide fragments of proinsulin, serve as potent antigenic ligands for multiple diabetogenic T cell clones.

More recently, we found that post-translational modification involving the B-chain of insulin, instead of C-peptide, results in the formation of the 6.3HIP (10), a B-chain HIP that when assayed as an antigenic peptide *in vitro*, is about ten thousand times stronger for our insulin-reactive diabetogenic T cell clone BDC-6.3 than the native insB_9–23_ sequence. The 6.3HIP is the hybrid peptide product of insB_14–19_ combined with ProSAAS_219-225_. Predictive modeling based on mutational analysis and peptide truncation studies placed the 6.3HIP within the insulin B_9–23_ register 3, and the fusion of proSAAS with insB_14–19_ allowed pocket 9 of I-A^g7^ to be occupied by an aspartic acid (D), a negatively charged amino acid, thereby providing a strong binding residue for this disease-critical pocket (10). The BDC-6.3 T cell clone responds to the 6.3HIP down to the low nanomolar range, thereby offering a possible explanation of how post-translational modification of the insulin B-chain can trigger strong T cell responses by a diabetogenic, islet-infiltrating CD4 T cell clone. We and others have isolated panels of diabetogenic CD4 T cell clones either from lymphoid organs of diabetic NOD mice (11, 12), or in the case of Wegmann et al., directly from the islets of 12-week-old pre-diabetic NOD mice (13). The majority of CD4 T cell clones isolated by the Wegmann group respond to insB_9-23_ (13), and require high concentrations (~micromolar) of peptide for robust T cell stimulation. One clone from the Wegmann PD panel, PD12-4.4, responds to the 6.3HIP much more strongly than to the native insB_9–23_ sequence (10).

The goal of our studies is to determine whether insulin-reactive CD4 T cell clones react to B-chain HIPs derived from potential CatD cleavage sites, and specifically, whether B-chain HIPs are strong agonists for these CD4 T cell clones and polyclonal T cells from NOD mice. We report here on the identification of a new B-chain HIP, termed the 2.40HIP, that is targeted by PD12-2.40, another diabetogenic CD4 T cell clone from the Wegmann panel, for which the native insulin sequence is also poorly antigenic. The 2.40HIP is formed by covalent cross-linking of the insulin B-chain on the N-terminal side with the C-peptide of proinsulin on the C-terminal end, resulting in a strong ligand for the PD12-2.40 T cell clone; moreover, the 2.40HIP is formed through proteolytic activity of cathepsin D. Finally, epitope mapping of the 2.40HIP identifies a new antigenic register within the insB_9–23_ region, an important step in understanding why different regions of insulin B-chain are antigenic for pathogenic CD4 T cells.

## Materials and methods

### Reagents and enzyme preparation for cathepsin D-mediated proteolysis of Insulin B-chain

Human insulin B-chain (GenScript, NJ, USA) was reconstituted to a concentration of 1 mM in HPLC grade H2O. Recombinant Mouse cathepsin D (MuCatD; Cat # 50127-M08H; Sino Biological, China) was diluted to a stock concentration of 1.82 μM in HPLC grade H_2_O. Serial dilutions of MuCatD were prepared to achieve final concentrations of 1.82 μM, 0.45 μM, 0.1125 μM, and 0.028 μM in the reaction mixtures. Concentrations of CatD were based on those previously optimized in experiments to investigate CatD cleavage sites in the C-peptide of proinsulin (8).

### Conditions for cathepsin D proteolysis of insulin B-chain

Reaction mixtures were assembled in microcentrifuge tubes, each comprising 10 μL of McIlvaine buffer (pH 4.0), 10 μL of 1 mM Human insulin B-chain, and 1 μL of the indicated enzyme dilution or buffer only (for control) for a final volume of 21 μL. Samples were incubated at 37 °C for 100 min.; reactions were terminated by addition of 21 μL of trifluoroethanol (TFE) to each tube and heat treatment at 70 °C for 10 min. Following reaction termination, samples were then lyophilized using a vacuum concentrator Integrated Speedvac (ThermoFisher Scientific, USA). Prior to mass spectrometry, lyophilized samples were reconstituted in 200 μL of solution containing 3% acetonitrile, 0.1% formic acid in HPLC-grade water. To ensure homogeneous resuspension, samples were sonicated for 5 minutes (Ultrasonic Cleaner, FS60H, Fisher Scientific), centrifuged for 5 minutes at 14,000 x g and supernatants were analyzed by mass spectrometry.

### Mass spectrometric analysis

Aliquots (5 μL) of each sample were transferred to autosampler vials for analysis by liquid chromatography-tandem mass spectrometry (LC-MS/MS). Mass spectrometric analysis was performed on an Agilent 6550 Q-TOF LC/MS system operating in positive ion mode. Raw data were extracted and analyzed using Spectrum Mill MS Proteomics Workbench (Rev. B.06.00.201, Agilent Technologies). Cleavage maps were generated by exporting B-chain peptide fragment spectral intensities from Spectrum Mill into Excel. For each peptide bond and cathepsin D concentration, a cleavage intensity was calculated by summarizing the observed spectral intensity of peptides ending and peptides starting at that bond. Measurements from 3 independent experiments were averaged to produce a composite cleavage profile for each cathepsin D concentration. This approach enabled direct comparison of cleavage patterns across the full range of enzyme concentrations.

### Mice

NOD (NOD/ShiLtJ (# 001976)) and NOD.*scid* (NOD.Cg-Prkdc*scid*/J (#001303)) mice were purchased from The Jackson Laboratory. NOD mice were monitored for development of diabetes by urine glucose testing (Diastix: Bayer); positive readings were verified by blood glucose testing with the OneTouch Ultra glucometer (LifeScan). Mice were considered diabetic when blood glucose levels exceeded 15 mmol/L (270 mg/dL) on two consecutive measures. Animals were housed in a pathogen-free environment and all experiments were conducted according to guidelines approved by the Institutional Animal Care and Use Committee at the University of Colorado School of Medicine.

### T cell clones and culture

Diabetogenic T cell clones BDC-4.38 Vβ6, BDC-4.38 Vβ12, and BDC-6.3 from the Haskins’ BDC panel were previously isolated and cloned from NOD mouse splenocytes and peripheral lymph nodes (11, 12). T-cell clones from the Wegmann panel (PD12-1.11, PD12-1.18, PD12-2.40, PD12-2.70, PD12-4.19 and PD12-4.4) were originally isolated from 12-week-old prediabetic NOD mouse islets (13–15). All T cell clones were stimulated every 2 weeks as previously described (12) in fresh cultures with whole porcine insulin (Sigma-Aldrich), NOD.*scid* islets, or a murine β-cell extract (β-membrane) (16), as sources of antigen in the presence of IL-2 (2.5% EL-4 cell supernatant).

### Peptides

B-chain HIPs and control peptides (including 2.5HIP, insB_9-23_, insB_9–23 Y16A_ and HEL_11-25_) were obtained at a purity of greater than 95% (GenScript, NJ, USA), were dissolved in DMSO (Sigma; cat#D2650) and are listed in **Supplementary Tables 2**, **3**. To generate a B-chain HIP library of new antigen candidates we assigned the left portion of each HIP to B_9-16_ (SHLVEALY) ending in the target site of CatD within the insulin 2 B-chain. Keeping the B-chain left peptide constant, C-terminal (right) peptides were selected based on ranked mass spectrometry abundance profiles of proteins from NOD and/or BALB mouse islets (7). Secretogranule proteins, which are typically proteolytically processed at dibasic cleavage sites, were selected containing an acidic residue four amino acids from the N-terminus of each peptide to optimally accommodate pocket 9 (p9) of the I-A^g7^ binding groove. Thus, every B-chain HIP in the library contains a D or E residue within the right peptide that can potentially fit in I-A^g7^ p9 (17). Right peptides were derived from proproteins GRP78, 7B2, Carboxypeptidase E (CarbE), Insulin 2 C-peptide (C-pep), Secretogranin 2 (Scg2), Chromogranins A (ChgA) and B (ChgB) and Prohormone Convertase 2 (PC2); peptides were each linked to the left peptide B_9–16_ to comprise the synthetic B-chain HIP library (**Supplementary Table 1**).

### Antigen assays

T cell clones (2 x 10^4^ cells) were co-cultured with antigen-presenting cells (2.5 x 10^4^ peritoneal exudate cells) and synthetic peptides (GenScript, NJ, USA), for 48 hrs in 0.25 ml in 96-well plates. Interferon gamma (IFN-γ) secretion in the culture supernatant was measured *via* ELISA using anti-mouse IFN-γ (BD Pharmingen, USA) to coat ELISA plates, biotin-labeled anti-mouse IFN-γ (BD Pharmingen, USA), streptavidin-peroxidase (Sigma), and 2,2’-Azino-bis(3-ethylbenzothiazolin-6-sulfonic acid) diammonium salt for colorimetric detection. Absorption was quantified at 415 nm on a microplate reader (iMARK, BIORAD).

### *In vitro* production of the 2.40HIP by cathepsin D.

Experiments involving *in vitro* formation of the 2.40HIP mediated by Cathepsin D were adapted from Crawford et al (8). Briefly, human insB_1-30_ (GenScript, NJ, USA) and mouse ins2 C-pep_1-11_ (GenScript, NJ, USA) were each reconstituted in ultrapure H2O and then diluted in 1 mM NaOH to obtain a 1 mM peptide solution. Peptides were incubated with and without Recombinant Mouse Cathepsin D (0.01 μM; Sino Biological cat # 50127-M08H), at pH 4.0 for 4 hrs at 37°C in an acetate buffer (Ammonium Acetate/Acetic Acid buffer). Reaction products were then dried using a centrifugal vacuum concentrator (ThermoFisher, USA), resuspended in complete media, and titrated into antigen assays.

### ELISpot assays

ELISpot assays were performed using a mouse IFN-γ ELISPOT kit (BD Biosciences) and the AEC substrate set (BD Biosciences), with splenocytes isolated from NOD mice. Briefly, spleens of diabetic NOD mice (12–25 week-old) were harvested in 15 ml of PBS (Gibco, USA) and single cell suspensions were obtained using a 70 μm filter. Red blood cell lysis buffer (5 ml) (Sigma, UK) was added to remove red blood cells, cell suspensions were spun down at *300g*, and splenocytes were resuspended in ELISPOT media. In each well, 5 x 10^5^ splenocytes were incubated with 10 μM peptide or control antigens (2.5HIP, HEL_11-25_) on plates coated with the anti-IFN-γ capture antibody for 72 hr. The sequence of peptides used in ELISPOT assays is provided in **Supplementary Table 2**. Spots were visualized after incubation with the biotinylated anti-IFN-γ secondary antibody and anti-streptavidin HRP, followed by AEC substrate and chromogen on an ELISPOT reader (CTL, ImmunoSpot, USA). For each antigen tested the total spots from 5 x 10^5^ total splenocytes were counted.

## Results

### HIPs containing the insB_9–16_ sequence elicit an inflammatory response from NOD splenocytes

**Figure 1:**
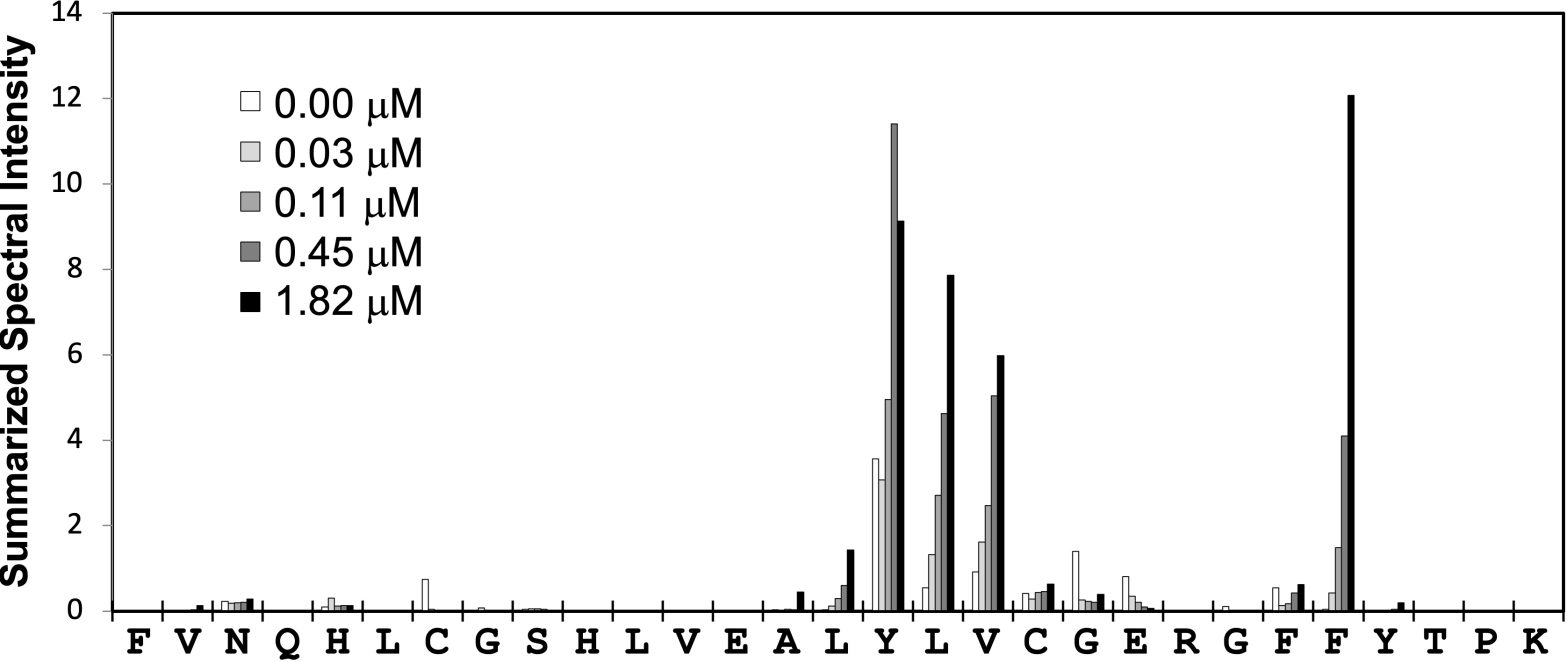
Mapping cathepsin D specificity for insulin B-chain. Insulin B-chain (100 μM) was incubated with CatD (0.03 μM, 0.11 μM, 0.45 μM or 1.82 μM) or without CatD, at pH 4.0 for 100 minutes at 37°C in McIlvaine Buffer. Samples were then analyzed by Agilent 6550 Q-TOF LC/MS system and raw data were extracted and analyzed using Spectrum Mill MS Proteomics Workbench. Each tick mark on the X-axis denotes an amino acid position, with adjacent positions representing peptide bonds. Spectral intensities for intact B-chain (insB_1-30_) are not shown. The height of vertical bars represents the summed spectral intensities of peptides cleaved at a particular amino acid bond, for each concentration of CatD. Data represent the average spectral intensities from 3 independent experiments.

Our previous work has demonstrated that cathepsin D mediates formation of HIPs, such as the 2.5 and 6.9HIPs, containing C-peptide sequences. To investigate whether CatD is also involved in formation of B-chain HIPs, wherein the N-terminal sequences are derived from insulin B-chain instead of C-peptide, the B-chain (insB_1-30_) of insulin was incubated with various concentrations of CatD *in vitro* and cleavage products of insB_1–30_ were assessed using mass spectrometry (**Figure 1**). We found that CatD cleaves insB_1–30_ at the C-terminal end of insB_F24_ residue, resulting in formation of the insB_1–24_ fragment. We also identified insB_1-15_, insB_1-16_, and insB_1–17_ as major products of CatD proteolytic action within the B_9–23_ region of the B-chain (**Figure 1**; **Supplementary Table 1**). Because the insB_Y16_ residue within the insB_9–23_ region is critical for disease pathogenesis (18) and because insB_1–16_ was identified as one of the major insulin fragments generated by CatD that retains this residue, we hypothesized that cleavage of the B-chain of insulin by CatD at the C-terminal of Tyrosine 16 (Y16) results in the formation of hybrid insulin peptides (HIPs) through a transpeptidation process and that these HIPs are ligands for islet-reactive CD4 T cells. To test this hypothesis, we constructed a peptide library of 24 B-chain HIPs (listed in **Supplementary Table 2**) containing insB_9–16_ as an N-terminal (left) peptide to screen splenocytes from diabetic NOD mice and assess T cell reactivity to insB_9–16_ HIPs in a polyclonal T cell setting.

**Figure 2:**
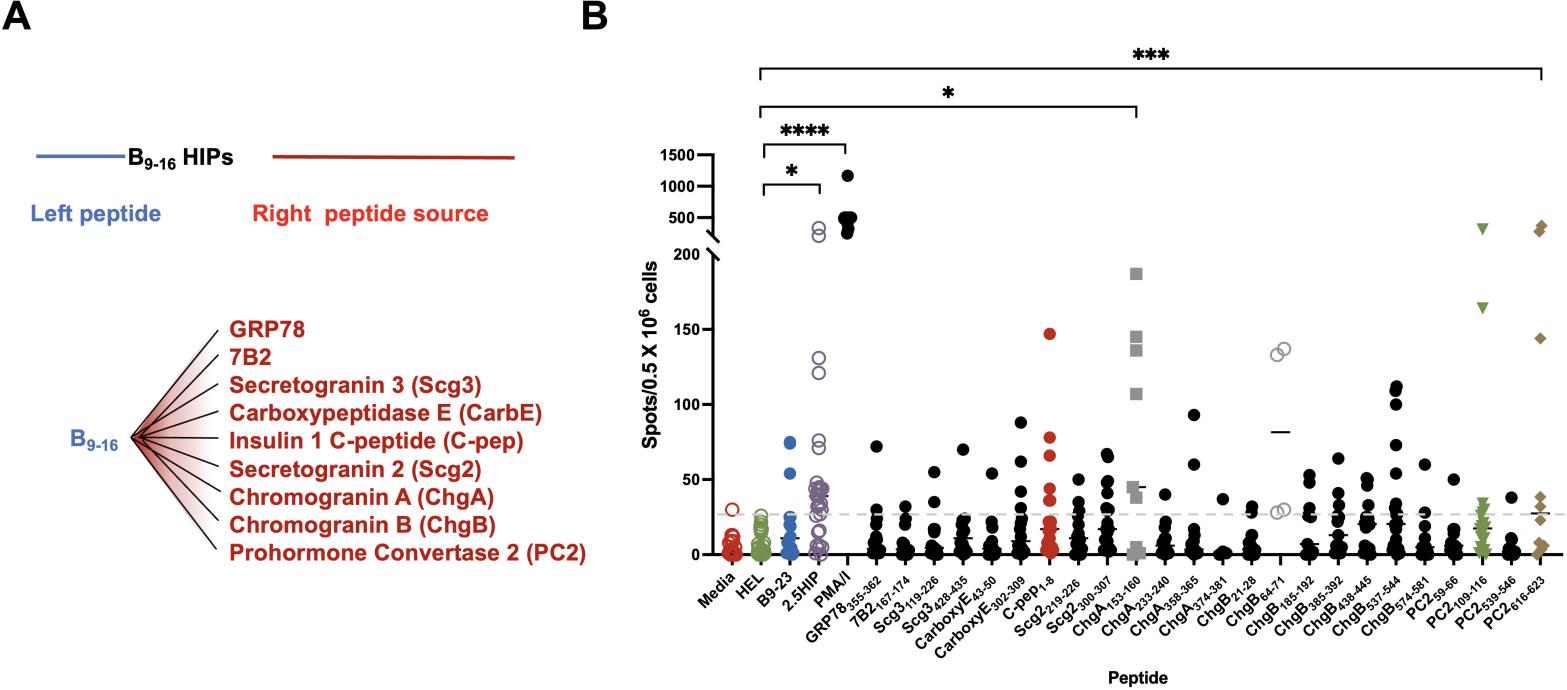
B-chain HIPs forming at the Y16 residue of the B-chain elicit an inflammatory response from splenocytes isolated from diabetic NOD mice. **(A)**. Schematics of B_9–16_ HIP library: B-chain HIPs contain the insB_9–16_ fragment on the left side (N-terminal) and 8 amino acids derived from other granule proteins on the right side (C-terminal). **(B)** Splenocytes from diabetic NOD mice were cultured with or without antigen (10 μM of peptide) or PMA/ionomycin for 72 hours in an IFN-γ ELISpot plate (BD Biosciences). IFN-γ-secreting cells are represented by the number of spots indicated on the Y-axis; each symbol represents an individual mouse with the horizontal dash representing the mean number of spots for each condition. Summary of 3 independent experiments, with each symbol representing one individual mouse, with a total of at least 8 mice tested per peptide condition. X-axis lists right peptides that extend 8 amino acids from proteins listed linked to ins2 B_9-16_. Gray dotted line represents the mean of response to HEL + 2xSD. *P<0.05, ***P=.0005, ****P<0.0001 compared to HEL in a one-way ANOVA test.

IFN-γ ELISPOT assays can indicate numbers of antigen-specific T cells present at very low frequencies in tissue or peripheral blood. We previously reported that compared to T cells reactive to the disease-relevant peptide (insB_9-23_), or to an irrelevant peptide antigen derived from Hen Egg Lysosyme (HEL_11-25_), frequencies of IFN-γ-secreting 2.5HIP-reactive T cells are significantly elevated in the spleens of diabetic NOD mice and correlate with cell numbers observed by tetramer staining using flow cytometry (19). Here we used ELISPOT to investigate the presence of T cells reactive to insB_9–16_ HIPs, in which assay a polyclonal population of diabetogenic T cells isolated from splenocytes of diabetic NOD mice (12–25 week-old) were stimulated with a panel of 24 insB_9–16_ HIPs. Our results indicate that whereas responses to the native B_9–23_ peptide were not significantly elevated compared to HEL_11-25_ (p>0.05), two insB_9–16_ HIPs (B_9-16_/PC2_616–623_ and B_9-16_/ChgA_153-160_) elicited significantly elevated IFN-γ responses from splenic T cells when compared to HEL_11-25_ (**Figure 2**). The PMA/ionomycin positive control also induced strong responses (p=0.0001). These results demonstrate that T cells reactive to insB_9–16_ HIPs are present in the polyclonal T cell population present in the spleen of diabetic NOD mice.

### The 2.40HIP is a strong agonist for the diabetogenic T cell clone PD12-2.40

**Figure 3:**
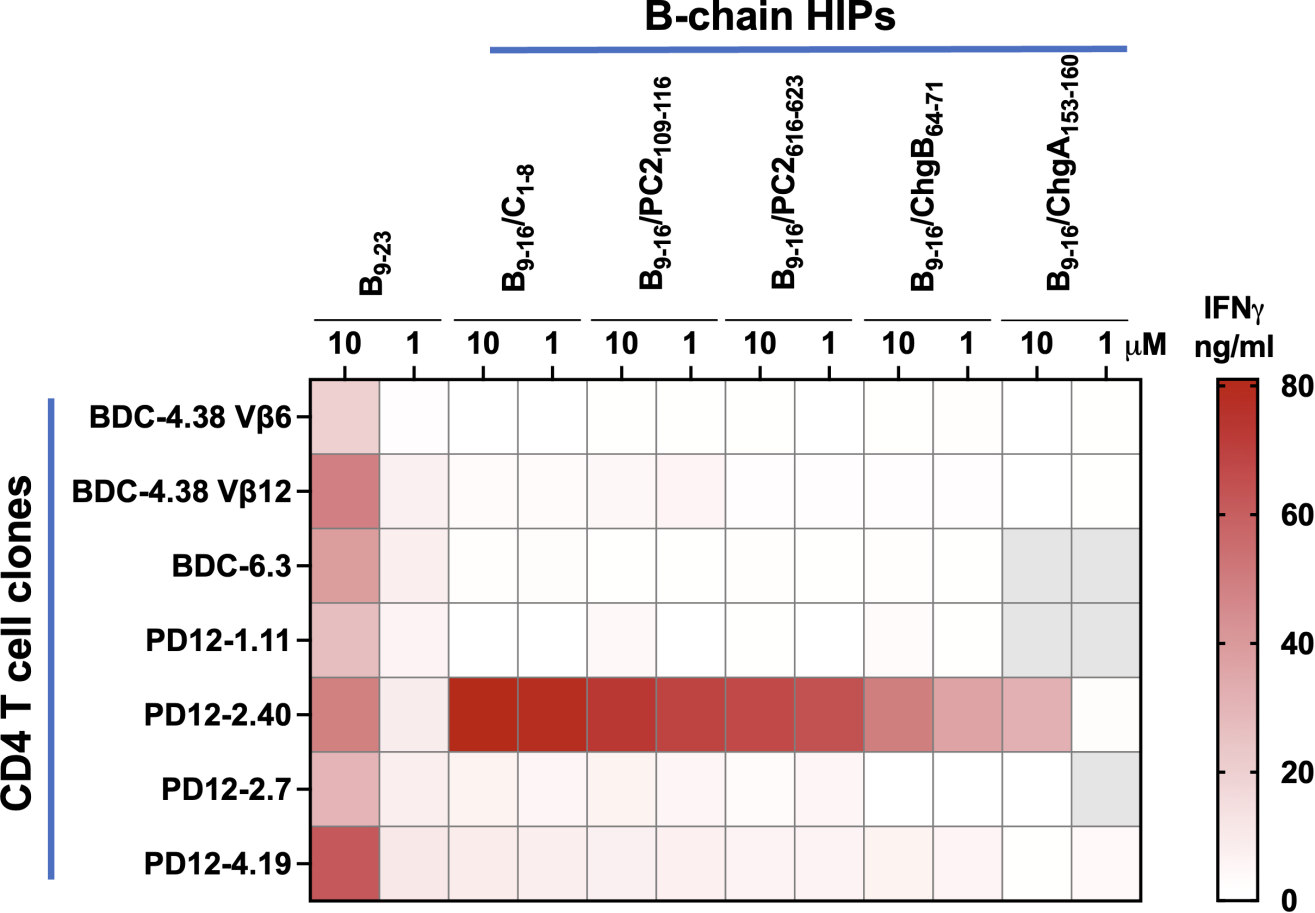
IFN-γ response of insulin-reactive T cell clones with B_9–16_ HIP candidates. CD4 T cell clones (2 x 10^4^) from the BDC or PD panel were challenged with peptides in the presence of peritoneal exudate cells (PECs; 2.5 x 10^4^) as antigen-presenting cells. After 48 hours, supernatants from cell culture wells were harvested and IFN-γ production was assessed by ELISA. Data are individual values from one representative experiment from 3 independent experiments. Ins2 B_9–23_ or B_9–16_ HIPs were used at a concentration of 10 and 1 μM. Heat map of IFN-γ (right) ELISA in ng/ml. Gray = not done.

To further characterize insB_9–16_ HIPs as ligands for diabetogenic T cells, we screened the peptide library of insB_9–16_ HIPs with insulin-reactive CD4 T cell clones from the BDC and PD panels. We first confirmed that all CD4 T cell clones listed in **Table 1** reacted to the native insB_9–23_ sequence and observed detectable T cell responses (**Figure 3**) at relatively high concentrations of peptide (10 μM). We then screened T cell reactivity to an extended panel of insB_9–16_ HIPs, which contained the 24 insB_9–16_ HIPs listed in **Supplementary Table 2** in addition to the insB_9-16_/GRP78_622–629_ HIP (which sequence is listed in **Supplementary Table 3**). **Supplementary Figure 1** is a heat map of the responses of two T cell clones, PD12-2.40 and BDC-4.38 Vβ12, to these HIPs. The T cell clone PD12-2.40, but not BDC-4.38 Vβ12, showed responses to several peptides in this panel of B_9–16_ HIPs, including HIPs containing right peptides from the following proteins: GRP78, insC, Scg2, PC2, ChgA and ChgB. (**Supplementary Figure 1**). Furthermore, of the seven insulin-reactive CD4 T cell clones in the panel, PD12-2.40 was the only T cell clone that showed responses to several peptides in this panel of B_9–16_ HIPs, including HIPs containing insC_1-8_, PC2_109-116_, PC2_616-623_, ChgB_64-71_ (**Figure 3**), GRP78_355–362_ and GRP78_622-629_ (**Supplementary Figure 1**) as right peptides. All these B-chain HIPs stimulated PD12-2.40 at 1μM and 10μM, except for the B-chain HIP containing the ChgB_64–71_ fragment to which there was a response at 10 μM only (**Figure 3**). None of the other insulin-responsive CD4 T cell clones listed in **Table 1** responded to any of the peptides present in the insB_9–16_ HIP library (**Figure 3**; **Supplementary Figures 2**, **3** and data not shown). Together, these findings demonstrate that multiple B-chain HIPs, partially derived from the CatD cleavage product insB_9-16_, can activate PD12-2.40, an insulin B-chain-reactive CD4 T cell clones.

**Table 1:** Insulin-reactive CD4 T cell clones.

T cell clone	B9–23 reactivity (10 μM)	Diabetogenic	TCR Vβ usage
BDC-4.38 Vβ6	+	+	Vβ6
BDC-4.38 Vβ12	+	+	Vβ12
BDC-6.3	+	+	Vβ1
PD12-1.11	+	nd	Vβ6
PD12-1.18	+	nd	Vβ6
PD12-2.40	+	+	Vβ8.3
PD12-2.70	+	nd	nd
PD12-4.19	+	nd	nd
PD12-4.4	+	+	Vb1

**Figure 4:**
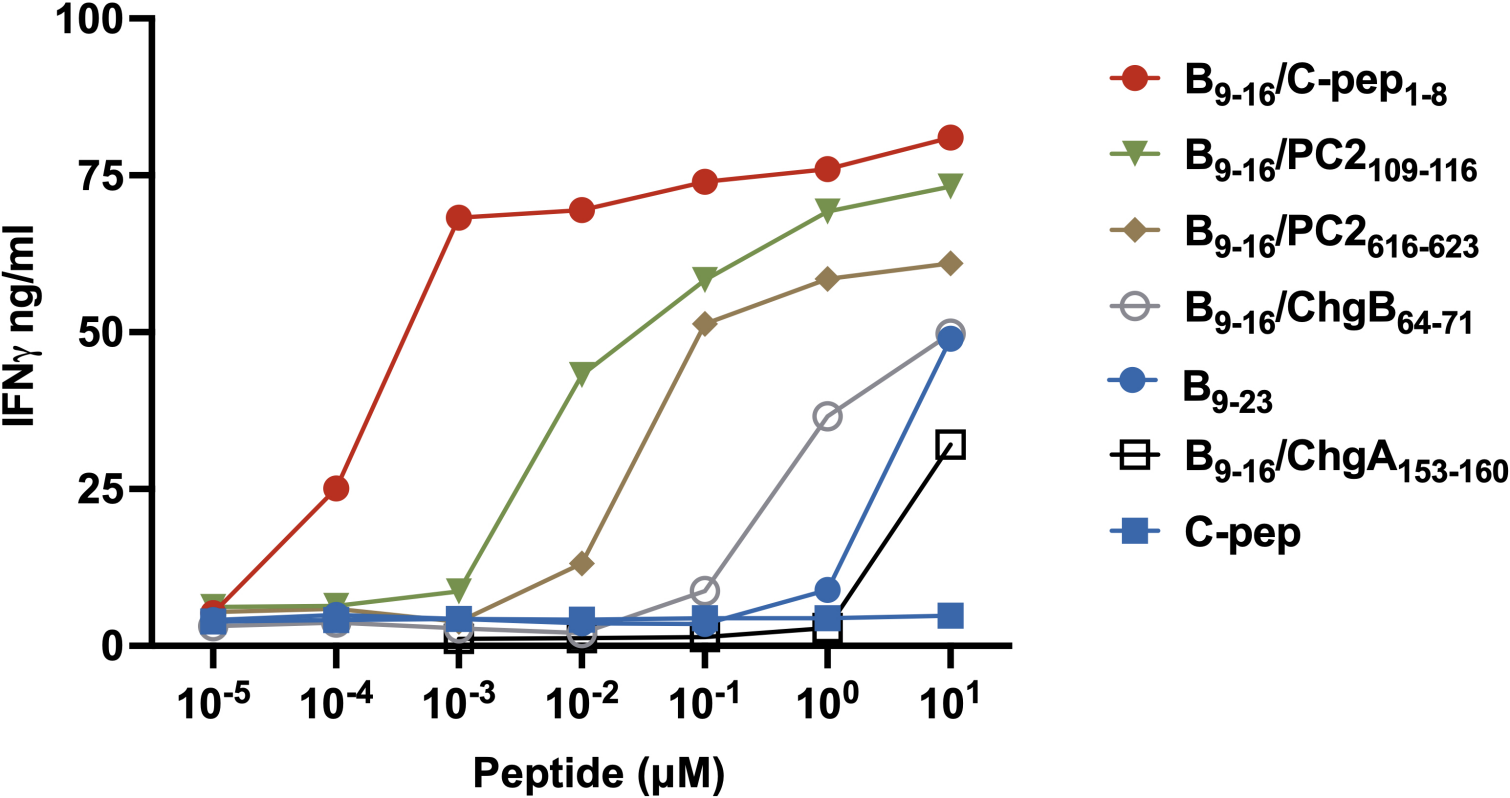
The PD12-2.40 diabetogenic T cell clone strongly responds to a B-chain/C-pep HIP. The PD12-2.40 CD4 T cell clone (2 x 10^4^) was challenged with peptides in the presence of PECs (2.5 x 10^4^). After 48 hours, IFN-γ production was assessed by ELISA. Data are individual values from one representative experiment and are representative of 3 independent experiments. Control peptides (ins2 B_9–23_ and ins2 C-peptide_1-11_) are shown in blue circles and squares respectively.

The cognate HIP antigens that have been identified for diabetogenic T cell clones BDC-2.5, BDC-6.9, NY4.1 and BDC-6.3 elicit responses at very low antigen concentrations, usually in the low nanomolar range. Since several peptides stimulated PD12-2.40 at 1 μM, these B-chain HIPs were further titrated in antigen assays to determine their potency. Our results shown in **Figure 4** demonstrate that the B-chain HIP containing insC_1–8_ at the C-terminal elicited the strongest response by PD12-2.40, with an EC50 close to 500 pM. PD12-2.40 also responded well to B-chain HIPs containing PC2_109–116_ and PC2_616-623_, whereas B-chain HIPs containing ChgB_64–71_ and ChgA_153–160_ fragments elicited responses only when peptide concentrations were above 1 μM. Since the insB_9-16_/insC_1–8_ B-chain HIP stimulated the PD12-2.40 T cell clone in the picomolar range, which is characteristic of a cognate peptide ligand for HIP-reactive T cells, we selected this B-chain HIP for further characterization and hereafter refer to it as the 2.40HIP.

### The diabetogenic T cell clone PD12-2.40 recognizes a novel register within the insB_9–23_ region

**Figure 5:**
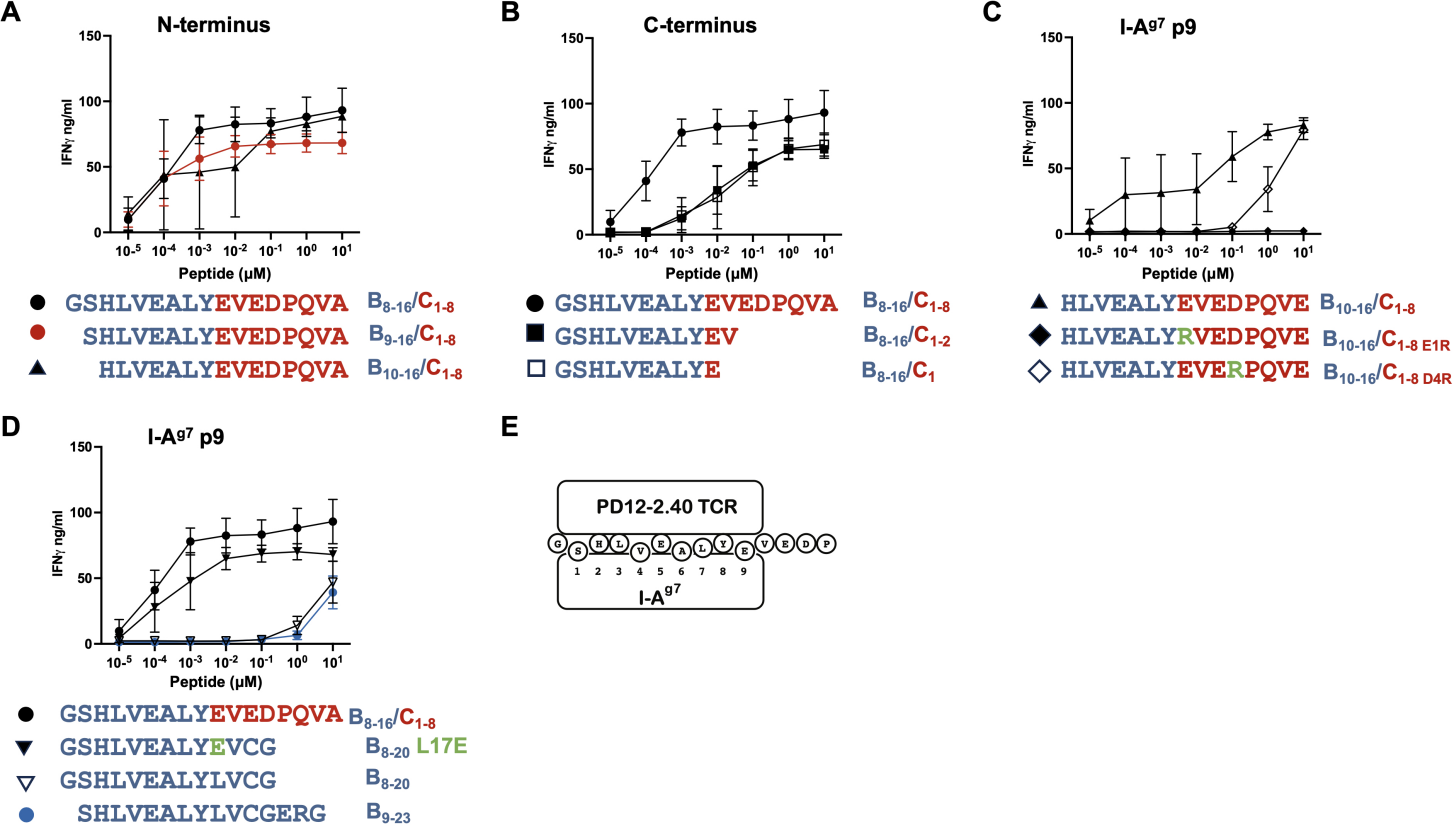
Epitope mapping of the 2.40HIP. The PD12-2.40 CD4 T cell clone (2 x 10^4^ cells) was challenged with peptides in the presence of PECs (2.5 x 10^4^). After 48 hours, supernatants from cell culture wells were harvested and IFN-γ production was assessed by ELISA. Data are representative of 3 independent experiments. B-chain sequences are shown in blue, C-peptide sequences are shown in red and point mutations in green. **(A)** Responses of PD12-2.40 to B_8-16_/C_1–8_ peptide (the 2.40HIP extended by one amino acid on the N-terminal) truncated at the N-terminal end. **(B)** IFN-γ response by PD12-2.40 to truncations of the B_8-16_/C_1–8_ peptide at the C-terminal end. **(C)** IFN-γ responses by PD12-2.40 to Arginine-mutated 2.40HIP. **(D)** Responses of PD12-2.40 to the B_9–23_ L17E mutated peptide or native sequences. Data are individual values from one representative experiment and are representative of 3 independent experiments. **(E)** Predicted binding of the 2.40HIP to I-A^g7^.

Epitopes presented by I-A^g7^, the NOD mouse MHC class II molecule, are typically 9 amino acids long and bind I-A^g7^ in pockets p1, p4, p6 and p9 (20). To define the core epitope for the 2.40HIP, the insB_8-16_/insC_1–8_ sequence was truncated at the N-terminal and purified peptides were used to stimulate PD12-2.40. Compared to insB_8-16_/insC_1-8_ (black circles), N-terminal truncation by one (insB_9-16_/insC_1-8_; red circles) or two (insB_10-16_/insC_1-8_; solid triangle) amino acids slightly diminished production of IFN-γ over the titration range (**Figure 5A**). Using the extended left peptide (insB_8-16_) for the 2.40HIP, we defined the epitope boundary at the C-terminus (**Figure 5B**). Truncation of seven residues of the right peptide (B_8-16_/C_1_, open square) still resulted in strong stimulation of PD12-2.40 (EC50 = 50 nM). Our results also indicate that the insB_8-16_/insC_1–2_ B-chain HIP stimulated PD12-2.40 in the same range as insB_8-16_/insC_1_, indicating a minimal contribution of the valine from the C-peptide fragment (**Figure 5B**). We therefore defined the 9-mer insB_9-16_/insC_1_ as the minimal epitope stimulating PD12-2.40.

It is well-established that the p9 pocket of I-A^g7^ prefers for binding a negatively-charged amino acid, e.g., aspartic acid (D) or glutamic acid (E) (20). Since the truncation studies indicated that the minimal epitope for the 2.40HIP is insB_9-16_/insC_1_ which contains a negatively charged amino acid (E) at position insC_1_, we investigated whether the glutamic acid provides an anchor residue for the p9 of I-A^g7^ by substituting the E with a positively charged arginine (R) residue (2.40HIP_E1R_). Our results (**Figure 5C**) indicate that this single amino acid substitution completely abrogated antigenic activity of the HIP, suggesting that insC_1_(E) interacts with the p9 pocket of I-A^g7^.

To further test whether the E residue interacts with the p9 pocket of I-A^g7^, we generated a mutant insB_8–20_ peptide containing a glutamic acid (E) at position 17 instead of a Leucine (L). **Figure 5D** shows that the resulting mutant peptide, insB_8–20 L17E_ (solid triangles), stimulated PD12-2.40 in a similar concentration range as the 2.40HIP (black circles) with an EC50 around 1 nM. The corresponding native sequence, insB_8-20_, had an EC50 above 10 μM (open triangles), indicating that mutation of Leucine 17 of the insulin B-chain into a negatively charged amino acid provided a strong anchor residue for the p9 pocket of I-A^g7^. Based on these findings, we propose a predictive model shown in **Figure 5E**, which would place the disease-critical Y16 amino acid of the insulin B-chain in position 8, a TCR contact residue (20, 21). Compared to the native B-chain sequence, we show that mutation of this amino acid Y16 to an alanine (insB_9–22 Y16A_) completely abrogates the stimulatory potential of the insB_9–22 Y16A_ peptide (**Supplementary Figure 3**).

### Cathepsin D mediates the formation of the 2.40HIP *in vitro*

**Figure 6:**
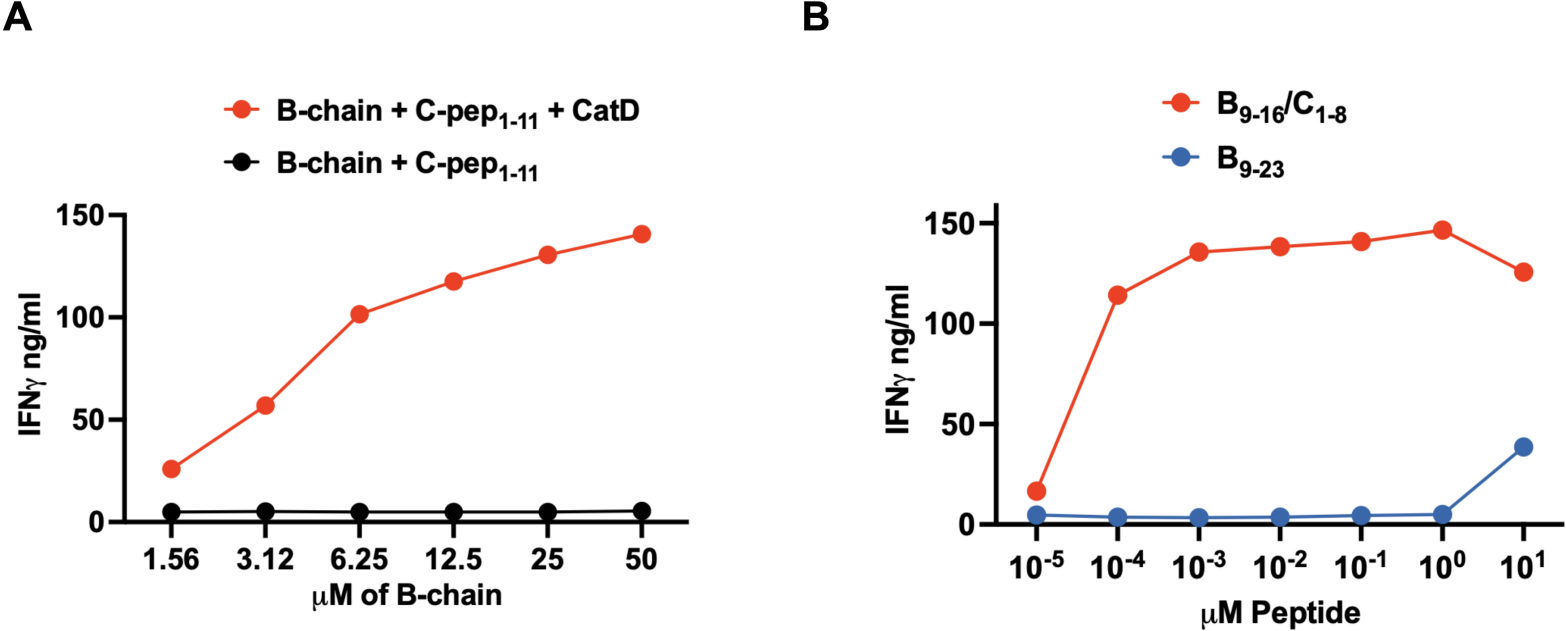
CatD mediates the formation of the 2.40HIP *in vitro*. The PD12-2.40 CD4 T cell clone (2 x 10^4^ cells) was cultured with PECs (2.5 x 10^4^) and challenged with the following peptide/CatD mixtures: **(A)** B-chain and C-peptide incubated with (red) or without (black) CatD; **(B)** insB_9-23_ (blue) and insB_9-16_/C_1-8_ (2.40HIP; red) used at indicated concentration range (0.00001 - 10 μM). After 48 hours, supernatants from cell culture wells were harvested and IFN-γ production was assessed by ELISA. Data are individual values from one representative experiment and are representative of 2 independent experiments and expressed in ng/ml of IFN-γ.

Our data indicate that the insB_Y16-L17_ peptide bond of the insulin B-chain can be cleaved by CatD (**Figure 1**) and we previously reported that the protease CatD can mediate the formation of C-peptide HIPs (such as the 2.5HIP) through transpeptidation (8). To test the hypothesis that the 2.40HIP can be formed by a CatD-mediated transpeptidation reaction between the B-chain of insulin and C-peptide, we incubated insulin B-chain and C-peptide in the presence or absence of CatD *in vitro* and then tested the two mixtures for antigenicity with the PD12-2.40 T cell clone. Our data show PD12-2.40 was strongly stimulated when the insulin B-chain and C-peptide were incubated in the presence of CatD (**Figure 6A**), but not when CatD was absent (**Figure 6A**), indicating that the formation of the 2.40HIP is CatD-dependent. Titration curves indicate confirms that CatD can mediate the formation of the 2.40HIP *in vitro*. Reaction mixtures containing CatD with insulin B-chain or CatD with C-peptide was used as a control and did not stimulate PD12-2.40 (**Supplementary Figure 4**). As a positive control, we tested the 2.40HIP obtained from a commercial source, which elicited a strong response by PD12-2.40 (**Figure 6B**). Collectively these data indicate that CatD can enzymatically mediate the formation of the 2.40HIP *in vitro*.

## Discussion

Here we report on the identification of a new region of the B-chain of insulin targeted by diabetogenic T cells isolated from NOD mice. ELISPOT data indicated that post-translational modification though transpeptidation of insB_9-16_ (produced by CatD cleavage of the B-chain) with other secretory granule proteins leads to the formation of potent T cell epitopes recognized by T cells present in the spleens of diabetic NOD mice. Moreover, using PD12-2.40, a diabetogenic T cell clone isolated from the islets of a 12-week-old NOD mouse, we demonstrate that fusion of the C-peptide with the insB_9–16_ fragment results in the formation of a hybrid insulin peptide that is about 10,000 times more stimulatory than the native insB_9–17_ peptide. We further show that the synthesis of this antigen, defined as the 2.40HIP, can be mediated by the protease cathepsin D. The presence of high levels of C-peptide in β-cells, as well as a high specificity of CatD for the cleavage of the B-chain of insulin between Y16 and L17, provide the ideal environment for the formation of the 2.40HIP.

Defining the different regions of the B-chain of insulin that are targeted by islet-reactive T cells is an important step in understanding the role of insulin as a trigger of autoimmune diabetes and may lead to additional epitopes that could be used in strategies to induce antigen-specific tolerance. The insB_12-20_, insB_13–21_ and insB_15–23_ epitopes have been well-defined by several groups (2). The unfavorable register insB_14-22_, which would place an arginine residue in p9 of I-A^g7^, requires the introduction of a different amino acid at residue 22 of the B-chain, either through a post-translational modification such as HIP formation (10) (6.3HIP) or through mimotope design (22) (insp8G and insp8E). In this study, we demonstrate that a new region of the B-chain of insulin, insB_9-17_, is recognized by a diabetogenic CD4 T cell clone isolated from the islet of a 12-week-old NOD mouse. The PD12-2.40 T cell clone also recognized several other B_9–16_ HIPs, including B-chain HIPs (containing sequences from PC2 and ChgA) that elicited significantly elevated inflammatory responses by splenocytes isolated from diabetic NOD mice. We have demonstrated that ChgA-deficient NOD mice are protected from autoimmune diabetes (5) and that ChgA can be the source of peptides found in disease-relevant C-peptide HIPs, such as the 2.5HIP (4, 23), and also a B-chain HIP, as shown in this study. Our previous work has shown that the C-peptide is an important source of sequences for HIP epitopes, either as left peptides (4) (2.5HIP and 6.9HIP), right peptides (2.40HIP) or both left and right peptides (9, 24) (HIP11). Because the C-peptide is not hormonally active, modification of its sequence through genetic manipulation (25) is possible without affecting normal β-cell function. For example, the negatively charged N-terminal Glutamic Acid of the C-peptide (EVED) provides a strong anchor residue for p9 of I-Ag7 for both HIP11 and 2.40HIP and could be targeted by a point mutation using CRISPR/cas9 to investigate whether the absence of HIPs containing a C-peptide fragment on the right side would delay or prevent disease progression in NOD mice.

In summary, this study sheds new light on an enduring question in T1D: how does insulin, a vital hormone that regulates blood glucose levels, become the target of islet-reactive T cells in the context of susceptible MHC alleles? Whereas the native insB_9–17_ sequence has been found in the immunoproteome (2) eluted from I-A^g7^, indicating that this is a naturally processed peptide, this sequence is poorly stimulatory for PD12-2.40, as well as for other insulin-reactive T cell clones. However, post-translational modifications that introduce a negatively charged residue within the insB_9–23_ region may create a strong anchor for binding at the p9 pocket of I-A^g7^, enhancing affinity relative to the native B-chain sequence. This register would also suggest that the disease-critical Y16 amino acid could be a TCR-contact residue in the p8 position of I-A^g7^. Overall, these results suggest that the PD12-2.40 T cell clone is stimulated by a previously undescribed register in the B-chain of insulin. The model in **Figure 5E** is based on mutation and truncation studies but additional X-ray crystallography would be necessary to formally demonstrate how the 2.40HIP is presented in the context of I-A^g7^.

## Data Availability

The original contributions presented in the study are included in the article/**Supplementary Material**. Further inquiries can be directed to the corresponding author.
