# Supplementary material for: A novel region of the insulin B9–23 sequence is targeted by a HIP-reactive diabetogenic T cell clone
Source: Front Immunol. 2026 Jul 29;17:1837267. doi: 10.3389/fimmu.2026.1837267 (PMC13462851; doi:10.3389/fimmu.2026.1837267)
Supplement: Supplementary file 1 [file DataSheet1.pdf]

		F	V	N	Q	H	L	C	G	S	H	L	V	E	A	L	Y	L	V	C	G	E	R	G	F	F	Y	T	P	K
0 uM CatD	Mean Spectral Intensities	0.006133	0.232518	0.001684	0.096502	0	0.738955	0.014311	0.039868	0.002613	0.012041	0.01079	0.003311	0.026323	0.031442	3.560348	0.549167	0.915453	0.413533	1.401534	0.809216	0.012597	0.109921	0.549222	0.045847	0.002071	0	0.000199	0	0.002025
	Standard Deviation	0.00756	0.229307	0.00194	0.090952	0	1.137637	0.018069	0.051048	0.004526	0.019564	0.015944	0.005414	0.023321	0.041785	3.548619	0.556711	1.134683	0.559301	2.134173	1.072417	0.017105	0.173583	0.812702	0.078045	0.003587	0	0.000345	0	0.003507
0.03 uM Cat D	Mean Spectral Intensities	0.004678	0.179873	0.002098	0.301202	0	0.040648	0.071967	0.049696	0.001983	0.005678	0.005119	0.000699	0.018116	0.121045	3.06837	1.318643	1.615325	0.28198	0.256037	0.344336	0.007364	0.005157	0.131	0.421184	0.006767	0	0.000631	0	0
	Standard Deviation	0.00574	0.167366	0.002644	0.12467	0	0.070404	0.098267	0.032658	0.003435	0.009834	0.007003	0.00121	0.017243	0.188179	2.369337	1.171462	1.948489	0.30224	0.255335	0.318783	0.011294	0.006764	0.122197	0.54291	0.011238	0	0.001093	0	0
0.11 uM CatD	Mean Spectral Intensities	0.005935	0.194278	0.001522	0.122186	0	0	0.019589	0.049396	0.002214	0.002587	0.010201	0.000384	0.038409	0.297006	4.950198	2.706621	2.471554	0.435188	0.231442	0.204531	0.005609	0.004037	0.177059	1.48105	0.010018	0	0.00023	0	0.001362
	Standard Deviation	0.007891	0.182073	0.001627	0.050498	0	0	0.017	0.037894	0.003834	0.004482	0.016852	0.000666	0.053663	0.376559	2.187173	1.998134	1.715807	0.50603	0.256202	0.262046	0.00818	0.005515	0.203058	1.479415	0.0119	0	0.000398	0	0.00139
0.45 uM CatD	Mean Spectral Intensities	0.026735	0.200577	0.001464	0.131782	0.00138	0	0.010652	0.042975	0.003211	0.008417	0.003001	0.000195	0.03044	0.604715	11.41153	4.620133	5.043691	0.456989	0.204477	0.097023	0.003408	0.003643	0.424976	4.100293	0.043966	0	0.00016	0	0.001446
	Standard Deviation	0.029691	0.255628	0.001374	0.111188	0.002391	0	0.005391	0.021456	0.005561	0.013304	0.004201	0.000337	0.02607	0.3674	5.178854	1.402089	0.472329	0.518634	0.160878	0.086862	0.004068	0.003684	0.378835	2.64054	0.036022	0	0.000277	0	0.002505
1.82 uM CatD	Mean Spectral Intensities	0.13291	0.285858	0.004048	0.134609	0	0.00078	0.012083	0.016796	0.001681	0.009261	0.004733	0	0.445503	1.42905	9.129283	7.8669	5.980292	0.628514	0.388053	0.064741	0.00112	0.00547	0.621915	12.07633	0.198597	0	0	0	0.004872
	Standard Deviation	0.144228	0.289795	0.004978	0.135347	0	0.001351	0.006356	0.015402	0.002912	0.008985	0.00565	0	0.210611	0.251708	1.525365	1.322628	2.514152	0.826809	0.232185	0.052937	0.001103	0.006062	0.379702	11.38669	0.144365	0	0	0	0.00489

Supplementary Table 1. Mean spectral intensities (blue rows) and Standard Deviation (clear rows) of insulin B-chain cleavage products at the C-terminal cleavage sites for each cathepsin D concentration. Data are expressed in arbitrary units.

Left sequence insB9-16	Right sequence	Protein	Amino acid position	Source
SHLVEALY	SDIDEIAL	GRP78	355-362	GenScript
SHLVEALY	LLYEKMKG	7B2	167-174	GenScript
SHLVEALY	LIDEYDST	Secretogranin 3	119-226	GenScript
SHLVEALY	GNKEDYDL	Secretogranin 3	428-435	GenScript
SHLVEALY	LQQEDGIS	Carboxypeptidase E	43-50	GenScript
SHLVEALY	NDDDSSFV	Carboxypeptidase E	302-309	GenScript
SHLVEALY	EVEDPQVE	C-peptide	1-8	GenScript
SHLVEALY	ESKDQLSE	Secretogranin 2	300-307	GenScript
SHLVEALY	ERVDEEQK	Secretogranin 2	219-226	GenScript
SHLVEALY	KDSDKGQQ	Chromogranin A	153-160	GenScript
SHLVEALY	AKQEEKEE	Chromogranin A	233-240	GenScript
SHLVEALY	WSRMDQLA	Chromogranin A	358-365	GenScript
SHLVEALY	LEGEDDPD	Chromogranin A	374-381	GenScript
SHLVEALY	APVDNRDH	Chromogranin B	21-28	GenScript
SHLVEALY	SGKEVKGE	Chromogranin B	64-71	GenScript
SHLVEALY	HIEDSGEK	Chromogranin B	185-192	GenScript
SHLVEALY	NHPDSELE	Chromogranin B	385-392	GenScript
SHLVEALY	LLDEGHYP	Chromogranin B	438-445	GenScript
SHLVEALY	GNPDDSFL	Chromogranin B	537-544	GenScript
SHLVEALY	PFSEDVNW	Chromogranin B	574-581	GenScript
SHLVEALY	LPFAEGLY	Prohormone Convertase 2	59-66	GenScript
SHLVEALY	GYRDINEI	Prohormone Convertase 2	109-116	GenScript
SHLVEALY	PRDDDSKV	Prohormone Convertase 2	539-546	GenScript
SHLVEALY	QELEEEEL	Prohormone Convertase 2	616-623	GenScript

Supplementary Table 2. Insulin $B_{9\text{-}16}$ HIP Library. All left peptides (blue) are insulin 2 B-chain amino acids selected from the preferred cleavage products of proinsulin by CatD (ins2 $B_{9\text{-}16}$). All right peptides (red) are natural dibasic cleavage products of proteins from NOD and Balb/C islets (ending in KK, RR, RK, or KR) identified by mass spectrometry.

Peptide name	Extended Peptide name	Peptide sequence	Source	Solvant	Figure
**B-chain**	**Human insulin B-chain**	FVNQHLCGSHLVEALYLVCGERGFFYTPKT	GenScript	HPLC grade H2O	#1 & 6
**C-pep**	**ins2 C_{1-11}**	EVEDPQVAQLE	GenScript	HPLC grade H2O	#6
**2.5HIP**	**2.5HIP soluble**	RGGLQTLALWSRMDGGR	GenScript	UltraPure H2O	#2 & S3
**HEL**	**HEL11-25**	AMKRHGLDNYRGYSL	GenScript	UltraPure H2O	#2
**B9-23**	**ins2 B9-23**	SHLVEALYLVCGERG	GenScript	DMSO	#2-6 and S1-3
**insB9-22 Y16A**	**ins2 B9-22 Y16A**	SHLVEALALVCGER	GenScript	DMSO	S3
**ins1 B8-18**	**ins1 B8-18**	GPHLVEALYLV	GenScript	DMSO	S3
**ins2 B8-18**	**ins2 B8-18**	GSHLVEALYLV	GenScript	DMSO	S3
**ins2 B9-16/GRP78 622-629**	**ins2 B9-16/GRP78 622-629**	SHLVEALYELEEIVQ	GenScript	DMSO	S1
**B_{8-16}/C_{1-8}**	**ins2 B_{8-16}/ins2 C_{1-8}**	GSHLVEALYEVEDPQVA	GenScript	DMSO	#5
**B_{9-16}/C_{1-8}**	**ins2 B_{9-16}/ins2 C_{1-8}**	SHLVEALYEVEDPQVA	GenScript	DMSO	#5
**B_{10-16}/C_{1-8}**	**ins2 B_{10-16}/ins2 C_{1-8}**	HLVEALYEVEDPQVA	GenScript	DMSO	#5
**B_{8-16}/C_{1-2}**	**ins2 B_{8-16}/ins2 C_{1-2}**	GSHLVEALYEV	GenScript	DMSO	#5
**B_{8-16}/C_{1}**	**ins2 B_{8-16}/ins2 C_{1}**	GSHLVEALYE	GenScript	DMSO	#5
**B_{10-16}/C_{1-8}**	**ins2 B_{10-16}/ins1 C_{1-8}**	HLVEALYEVEDPQVE	GenScript	DMSO	#5
**$B_{10-16}/C_{1-8\ E1R}$**	**ins2 B_{10-16}/ins1 $C_{1-8\ E1R}$**	HLVEALYRVEDPQVE	GenScript	DMSO	#5
**$B_{10-16}/C_{1-8\ D4R}$**	**ins2 B_{10-16}/ins1 $C_{1-8\ D4R}$**	HLVEALYEVERPQVE	GenScript	DMSO	#5
**B_{8-20} L17E**	**ins2 B_{8-20} L17E**	GSHLVEALYEVCG	GenScript	DMSO	#5
**B_{8-20}**	**ins2 B_{8-20}**	GSHLVEALYLVCG	GenScript	DMSO	#5

Supplementary Table 3: Additional peptides used in the study with name of the manufacturer, sequence and Figure reference.

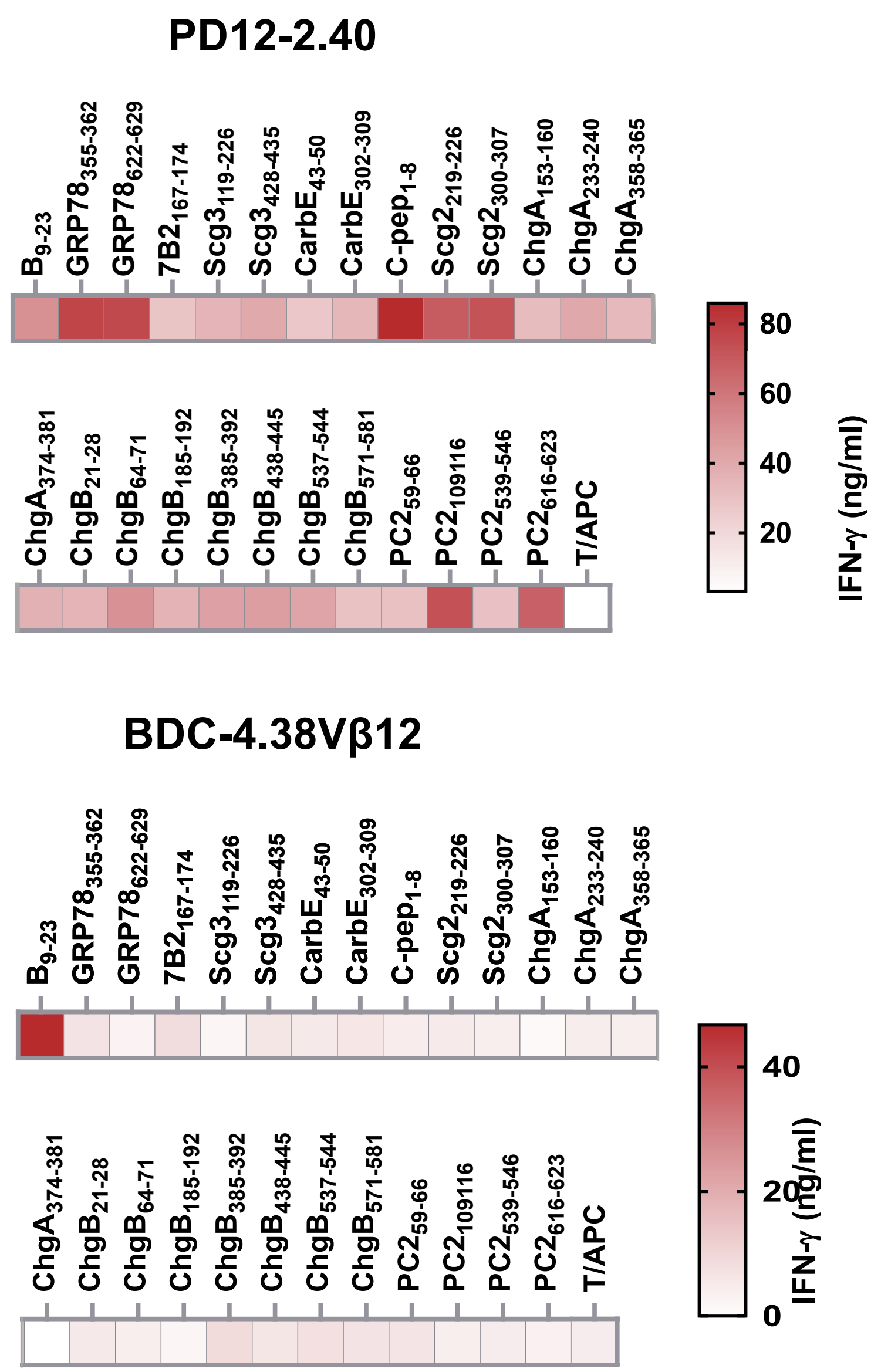

Supplementary Figure 1. Heat map of IFN-γ response of insulin-reactive T cell clone PD12-2.40 and BDC-4.38 Vβ12 with B$_{9-16}$ HIP antigen candidates. The PD12-2.40 and BDC-4.38 Vβ12 CD4 T cell clones were challenged with a panel of insB$_{9-16}$ HIP peptides or insB$_{9-23}$ at a concentration of 10 μM in the presence of PECs. B-chain HIPs contain B$_{9-16}$ on the left side and 8 amino acids derived from the secretogranule proteins on the right side (listed in Suppl. Table 2 & 3). Data from one experiment and are representative of four (BDC-4.38Vβ12) or five (PD12-2.40) experiments. IFN-γ expressed in ng/ml. T=T cells, APC=Peritoneal Exudate Cells as antigen presenting cells.

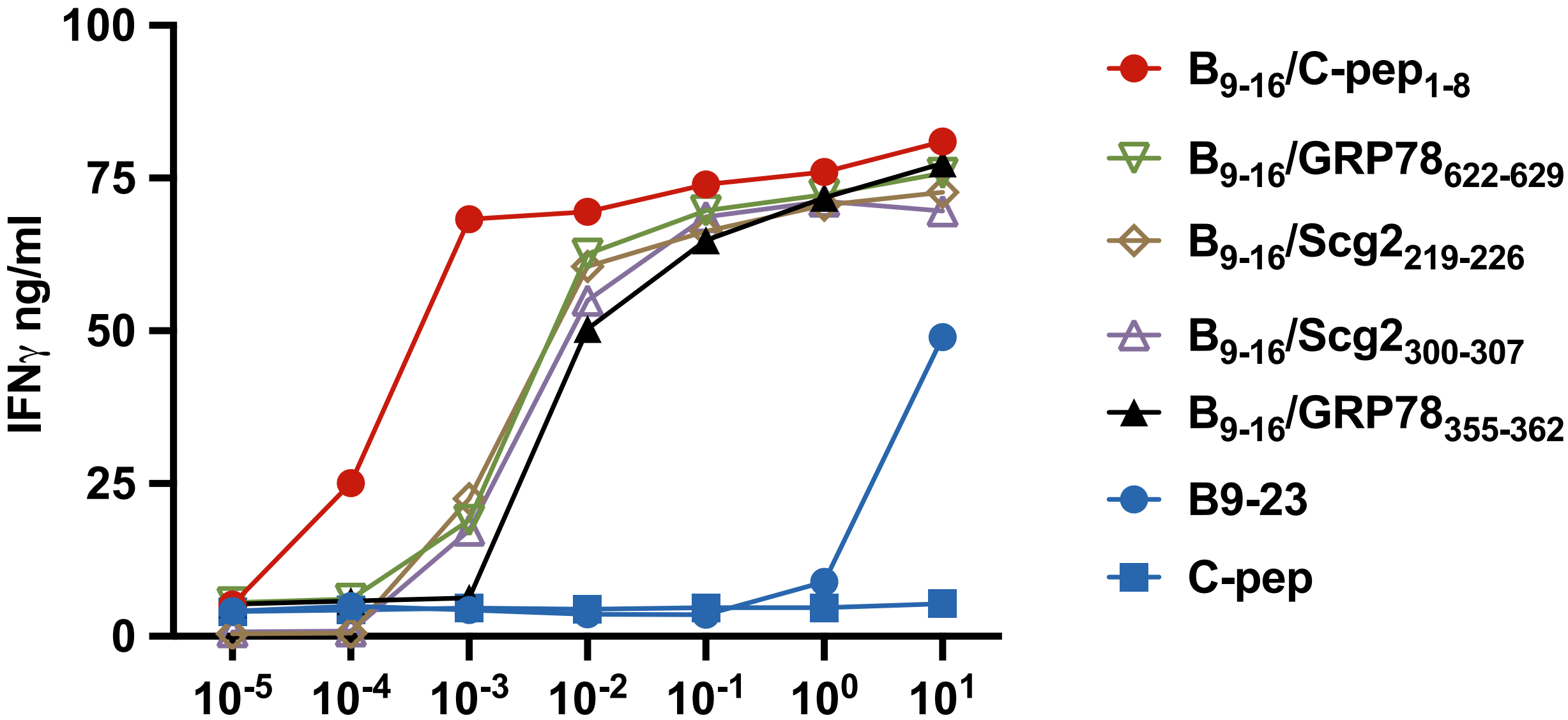

Supplementary Figure 2. IFN-γ production by T cell clone PD12-2.40 in response to titrations of $B_{9\text{-}16}$ HIPs. Data is shown in ng/ml IFN-γ and is the mean of three or more experiments. $B_{9\text{-}16}$ HIP sequences are listed in Suppl. Tables 2 & 3. Native sequences for control peptides are listed in Suppl. Table 3 and are ins2 $B_{9\text{-}23}$ (blue circles) and ins2 C-peptide$_{1\text{-}11}$ (blue squares).

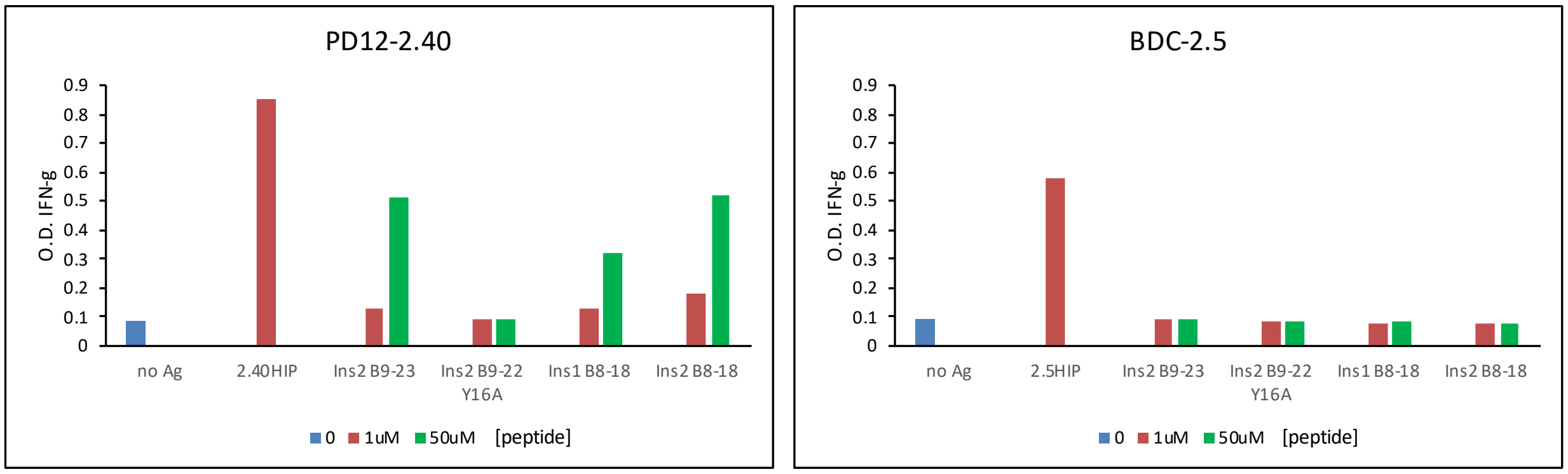

Supplementary Figure 3. Y16A mutation in the insulin B-chain abrogates response by PD12-2.40. PD12-2.40 or BDC-2.5 T cell clones were stimulated with the following peptides: 2.40HIP (ins2 $B_{9\text{-}16}/C_{1\text{-}8}$), 2.5HIP, ins2 $B_{9\text{-}23}$, ins2 $B_{9\text{-}22\ Y16A}$, ins1 $B_{8\text{-}18}$ or ins2 $B_{8\text{-}18}$ in the presence of antigen presenting cells (PECs). Peptides were tested either at 50 μM (green bars) or 1 μM (red bars). After 48h, supernatants from T cell cultures were tested for IFN-γ and average from duplicate wells are reported. Data indicate that the Y16 residue of the insulin B-chain is a critical residue and that ins1 $B_{8\text{-}22}$ and ins2 $B_{8\text{-}22}$ have comparable stimulatory activity. Data are from one experiment and are representative of 3 independent experiments. No Ag: T cells cultured with PEC only (no antigen; blue bars).

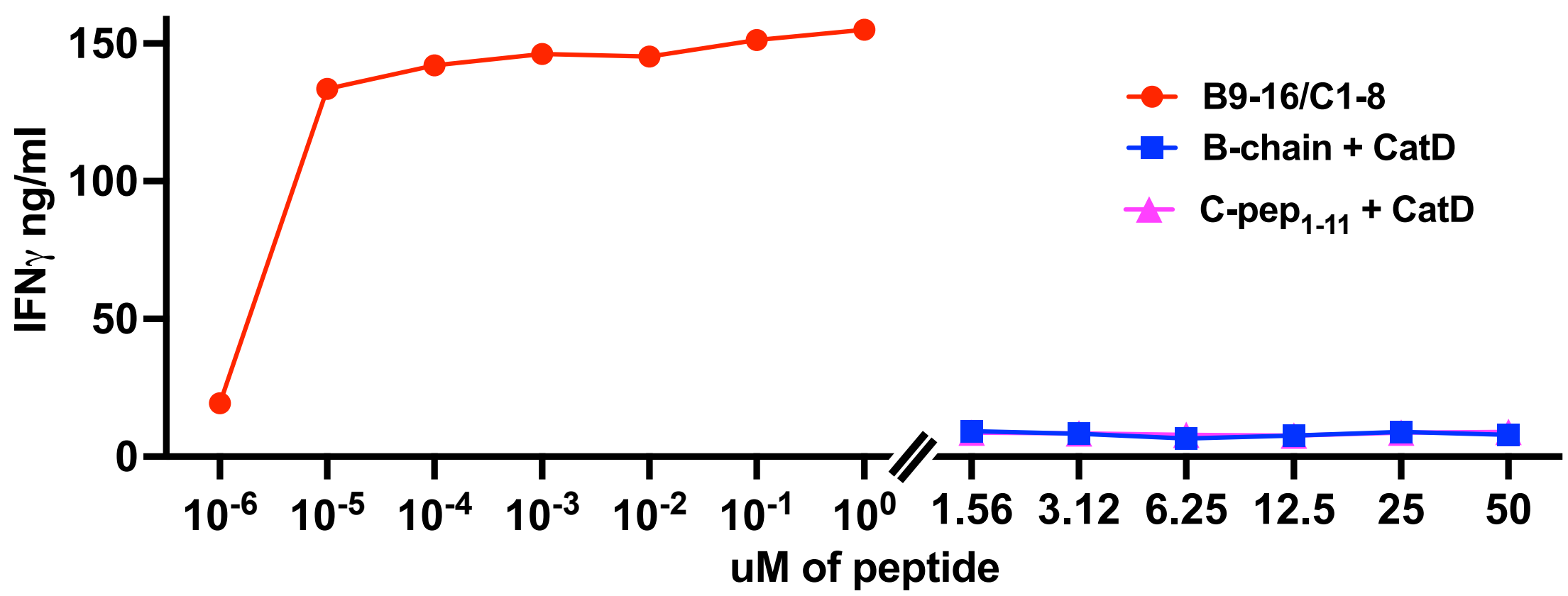

Supplementary Figure 4. The PD12-2.40 T cell clone does not react to CatD+C-peptide or CatD+B-chain. The PD12-2.40 CD4 T cell clone (2×10^4 cells) was cultured with PECs (2.5×10^4) and challenged with: 0.01 μM of CatD + C-pep_{1-11} (pink triangles) or 0.01 μM of CatD + ins B-$chain_{1-30}$ (blue squares) or with the 2.40HIP (ins2B_{9-16}/ins2C-$peptide_{1-8}$; red circles). Data are individual values from one experiment and are representative of 2 independent experiments expressed in ng/ml of IFN-γ.
